# Who meets national early childhood sleep guidelines in Aotearoa New Zealand? A cross-sectional and longitudinal analysis

**DOI:** 10.1093/sleepadvances/zpac002

**Published:** 2022-01-16

**Authors:** D Muller, E Santos-Fernández, J McCarthy, H Carr, T L Signal

**Affiliations:** 1 Sleep/Wake Research Centre, School of Health Sciences, College of Health, Massey University, Wellington, New Zealand; 2 Faculty of Science, School of Mathematical Sciences, Queensland University of Technology, Brisbane, Australia; 3 Ministry of Health, Wellington, New Zealand

**Keywords:** child/children, pediatrics, pediatrics—sleep and arousal, behavioral sleep medicine

## Abstract

**Study Objectives:**

To investigate the proportion of children in Aotearoa New Zealand (NZ) who do or do not meet sleep duration and sleep quality guidelines at 24 and 45 months of age and associated sociodemographic factors.

**Methods:**

Participants were children (*n* = 6490) from the *Growing Up in New Zealand* longitudinal study of child development with sleep data available at 24 and/or 45 months of age (48.2% girls, 51.8% boys; 22.4% Māori [the Indigenous people of NZ], 12.9% Pacific, 13.4% Asian, 45.2% European/Other). Relationships between sociodemographic factors and maternally reported child sleep duration (across 24 hours) and night wakings were investigated cross-sectionally and longitudinally. Estimates of children in NZ meeting sleep guidelines were calculated using a range of analytical techniques including Bayesian linear regression, negative binomial multiple regression, and growth curve models.

**Results:**

In NZ, 29.8% and 19.5% of children were estimated to have a high probability of not meeting sleep duration guidelines and 15.4% and 8.3% were estimated to have a high probability of not meeting night waking guidelines at 24 and 45 months respectively, after controlling for multiple sociodemographic variables. Factors associated cross-sectionally with children’s sleep included ethnicity, socioeconomic deprivation, material standard of living, rurality, and heavy traffic, and longitudinal sleep trajectories differed by gender, ethnicity, and socioeconomic deprivation.

**Conclusions:**

A considerable proportion of young children in NZ have a high probability of not meeting sleep guidelines but this declines across the ages of 24 and 45 months. Sleep health inequities exist as early as 24 months of age in NZ.

**Statement of Significance**

This research provides evidence that a substantial proportion of 24- and 45-month-old children in NZ have a high probability of not meeting sleep guidelines and that sleep health inequities exist as early as two years of age. A broad range of sociodemographic factors, including ethnicity, socioeconomic deprivation, and material standard of living, are associated with sleep in early childhood. Findings highlight the importance of tackling socio-political drivers of sleep inequities and taking a multi-level approach to supporting child sleep, as opposed to sleep interventions being solely focused on behavioral sleep advice at the individual level. Further work is required to elucidate mechanisms involved in sleep health inequities and relationships between neighborhood-, household- and child-level factors and sleep in early childhood.

## Introduction

Good sleep health in early childhood is crucial for supporting mental and physical health and development, including cognitive functioning, emotional and behavioral regulation, healthy body composition, and reducing injury risk [1–5]. Understanding how many children are meeting, and not meeting, sleep health guidelines and risk factors for poor sleep health is therefore vital for informing action to support all children to sleep well and flourish.

In Aotearoa/New Zealand (NZ) the Ministry of Health recommends that toddlers (1─2 year-olds) obtain 11 to 14 hours, and preschoolers (3─4 year-olds) obtain 10 to 13 hours, of good quality sleep across 24 hours each day [6]. These recommendations are based on US National Sleep Foundation (NSF) sleep duration guidelines [7] with the exception that preschoolers in NZ are defined as 3 to 4 years of age, as opposed to 3 to 5 years of age in the US, to reflect the fact that most children in NZ start school on or around their fifth birthday. Infants and toddlers in NZ (birth to 36 months; *n* = 1081) have been shown to have longer total sleep durations across 24 hours than in many other countries [8], although inequitable patterns of child sleep health have been identified within NZ [9, 10]. Recent research from a cohort of 340 Māori (the Indigenous people of NZ) and 570 non-Māori preschoolers indicates that short (<10 hours) and disturbed sleep is greater for Māori than non-Māori 3 to 4 year-olds and that socioeconomic deprivation is a significant independent risk factor that explains some but not all of the relationship between ethnicity and sleep [9, 10]. However less is known about whether inequities exist earlier in the life-course or for young children from other ethnic groups, and environmental and behavioral factors associated with meeting or not meeting sleep health guidelines in early childhood in NZ.

The current research utilized data from the *Growing Up in New Zealand* (GUiNZ) study [11, 12], which is the largest contemporary longitudinal study of child health and development in NZ, to address these identified knowledge gaps. Commencing in 2008, pregnant women living in three neighboring District Health Board areas (Auckland, Counties-Manukau and Waikato) in the North Island of NZ were recruited, resulting in a sample of 6846 children who were born between March 2009 and May 2010. Strengths of the GUiNZ study include the large sample size, longitudinal design, and ethnic and socioeconomic diversity of participants reflective of the NZ population [11, 12]. Access to GUiNZ data provided the opportunity to investigate sleep across early childhood within the GUiNZ cohort with the aim of (1) calculating estimates of children in NZ meeting and not meeting sleep duration and sleep quality guidelines at 24- and 45 months of age, both cross-sectionally and longitudinally; and (2) identifying sociodemographic factors associated with 24- and 45-month old children in NZ meeting or not meeting sleep duration and sleep quality guidelines.

## Methods

Ethics approval was granted for the GUiNZ study by the Ministry of Health Northern Y Regional Ethics Committee [11]. The current project was evaluated by peer review and judged to be low risk (Massey University Human Ethics Notification: 4000018816).

Sleep and sociodemographic data were collected from mothers in GUiNZ during face-to-face interviews when children were 24 months old, and brief telephone interviews when children were 45 months old.

### Sleep measures

Maternal report of children’s usual sleep duration at night (“On average, how much time does [child] spend asleep at night in total?”) and during the day (“On average, how much time does [child] spend asleep during the day?”) were summed to create total sleep time across 24 hours (*TST*). TST was categorized using NZ Ministry of Health sleep duration guidelines [6] to indicate if children had *“appropriate”, “short”* or *“long”* sleep durations. “Not recommended” short (age 1─2 years: <9 hours; age 3─4 years: <8 hours) and “may be appropriate” short (age 1─2 years: 9─10 hours; age 3─4 years: 8─9 hours) were collapsed to form the “*short*” category. “Not recommended” long (age 1─2 years: >16 hours; age 3─4 years: >14 hours) and “may be appropriate” long (age 1─2 years: 15─16 hours; age 3─4 years: 14 hours) were collapsed to form the “*long*” category.

Children’s night wakings (“On average how many times does [child] wake at night?”) were categorized using NSF sleep quality guidelines [13] to indicate “*appropriate*” (0─1), “*uncertain*” (2) or “*not appropriate*” (≥3) number of night wakings.

### Sociodemographic measures

Child *gender* (“Did you have a boy or a girl?”) was collected via telephone interviews at 6 weeks postpartum and child *ethnicity* was collected via face-to-face interviews with mothers when children were 9 months old (“What ethnic group does your baby belong to?” [mother was shown a list of ethnic groups and could select one or more ethnicities]). As per standardized ethnicity data protocols, we report prioritized ethnicity whereby each child was allocated to a single ethnic group, in prioritized order of Māori, Pacific, Asian, and European/Other [14].

Socioeconomic position was measured using the *NZDep2006* Index of Deprivation [15] at 24 months and the *NZDep2013* Index of Deprivation [16] at 54 months (*NZDep*), which was the closest point in time to the 45-month data collection. NZDep is an area-level composite measure of relative socioeconomic deprivation assigned to small geographical areas, based on eight dimensions of deprivation. NZDep quintiles were used in analyses (1 = least deprived through to 5 = most deprived).

Neighborhood-level environmental measures included *rurality* (urban or rural address), at 24 and 45 months; *neighborhood safety* (“This is a safe neighborhood”) dichotomized as disagree/agree, at 24 months; and *heavy traffic* (“There is heavy traffic on my street or road”) dichotomized as disagree/agree, at 24 months.

Household measures included *material standard of living* (“How does your [and your partner’s combined] total income meet your everyday needs?”) categorized as not enough money/just enough money/enough money/more than enough money, at 24 months; *household structure* (adults in child’s household) categorized as parent alone/two parents/parent(s) with others, at 24 and 45 months; *maternal paid work* (“Do you have a paid job at the current time?”) dichotomized as yes/no, at 24 and 45 months; and *childcare* (“Over the past 1 month has [child] been looked after at regular times during the week by anyone other than your partner?”) dichotomized as yes/no, at 24 months.

Child-level measures included time outside on weekdays (*time outside week*; “Thinking about the last 4 weeks, approximately how many hours has [child] spent outdoors on an average weekday?”) categorized as <1 hour and none/ 1 to <2 hours/ 2 to <3 hours/ 3 to <4 hours/ 4 to <5 hours/ ≥5 hours, at 24 months; *general health* (“In general how would you say [child’s] current health is?”) categorized as poor/fair/good/very good/excellent, at 24 months; *body size* (body mass index based on height and weight measurements taken during face-to-face interviews) categorized as thin/healthyweight/overweight/obese using International Obesity Taskforce BMI cut-offs [17], at 24 months); and *visual media use* (usual amount of time child spends using visual screen media on weekdays) categorized as <1 hour/ 1 to <2 hours/ 2 to <3 hours/ ≥3 hours, at 45 months.

### Statistical analysis

Data were analyzed using R software (version 3.4.3) [18]. Descriptive statistics were produced for sleep and sociodemographic variables, and percentages and 95% confidence intervals were calculated for categorical sleep variables stratified by categorical sociodemographic variables. Estimates of preschoolers in NZ meeting or not meeting sleep duration and night waking guidelines were calculated using Bayesian statistical modeling (Supplementary Material S1). Associations between sociodemographic variables and TST and night wakings at 24 and 45 months were investigated cross-sectionally using Bayesian multivariate linear regression and Bayesian negative binomial multiple regression models, controlling for all sociodemographic measures outlined above. The advantages of using a Bayesian paradigm of modeling include producing measures of uncertainty for estimated parameters and being able to model complex problems with numerous latent variables, unlike more traditional approaches [19–21].

A probabilistic estimate for each parameter was obtained from Bayesian models using Markov Chain Monte Carlo (MCMC) simulations. This enabled the estimation of the probability of each child falling below or above the sleep guidelines threshold to be calculated. We used non-informative normal priors for the regression coefficients with large variances, which had a minimum weight in the posterior distributions and inverse-gamma priors were used for the variances. Missing data were imputed by the model using posterior predictive distributions. We calculated the highest density intervals (HDI) in the posterior distributions, which represents the interval in which the latent parameters would lie with a certain probability, given the observed data. Sleep duration and night waking trajectories from 24 to 45 months were analyzed using Bayesian growth curve models adjusted for child gender, ethnicity, and NZDep quintile simultaneously. These growth curve models provide a flexible approach for analyzing longitudinal data accounting for change at both the group- and individual-level [22].

## Results

Sleep data were available for 6308 children at 24 months and 6186 children at 45 months. A total of 6490 children had sleep data available at one or more timepoints and 6004 children had sleep information at both timepoints. The total sample (*n* = 6490; 48.2% girls, 51.8% boys) comprised 22.4% Māori, 12.9% Pacific, 13.4% Asian and 45.2% European/Other children (6.1% missing ethnicity data). Nearly one quarter of children lived in areas with greatest socioeconomic deprivation (NZDep quintile at 24 months: 1 = 17.4%, 2 = 17.6%, 3 = 16.9%, 4 = 19.1%, 5 = 23.9%, missing data = 5.3%).

On average, children in the GUiNZ cohort slept over 12 and a quarter hours per 24 hour period at 24 months and approximately 11 and a quarter hours at 45 months of age, and the percentage of children waking more than once per night decreased from 24 to 45 months (Table 1). Approximately one quarter of children in the GUiNZ sample did not meet sleep duration and night waking guidelines at both timepoints (Table 2).

**Table 1.** Descriptive statistics describing sleep during early childhood for children in the GUiNZ cohort: cross-sectional sleep duration and night wakings at 24 and 45 months

Sleep variable	24 months (n=6308)	45 months (n=6186)
Night sleep duration (hours):		
Mean (SD)	10.55 (1.34)	10.69 (1.08)
Day sleep duration (hours):		
Mean (SD)	1.80 (0.79)	0.51 (0.85)
TST (hr):		
Mean (SD)	12.36 (1.51)	11.21 (1.18)
TST (%):		
Short^a^	11.86%	6.21%
Appropriate^b^	82.80%	90.61%
Long^c^	5.34%	3.18%
Number of night wakings (%):		
0	50.70%	62.01%
1	32.42%	30.86%
2	12.14%	5.64%
3 or more	4.74%	1.49%
Night wakings (%):		
Appropriate (0-1)	83.12%	92.87%
Uncertain (2)	12.14%	5.64%
Not appropriate (≥3)	4.74%	1.49%

^a^<11 hours at 24 months and <10 hours at 45 months;

^b^11-14 hours at 24 months and 10-13 hours at 45 months;

^c^>14 hours at 24 months and >13 hours at 45 months

**Table 2.** Contingency table of the proportion of children in GUiNZ following different sleep duration and night waking trajectories from 24 to 45 months

Sleep duration from 24 to 45 months (n=6004)			
	45 months:	45 months:	45 months:
	Short (<10 hours)	Appropriate (10–13 hours)	Long (>13 hours)
24mth:			
Short (<11hr)	2.03%	8.98%	0.35%
24mth:			
Appropriate (11-14hr)	3.91%	77.33%	2.12%
24mth:			
Long (>14hr)	0.15%	4.56%	0.57%
Night waking from 24 to 45 months (n=6004)			
	45 months:	45 months:	45 months:
	Appropriate (0–1)	Uncertain (2)	Not appropriate (≥3)
24mth:			
Appropriate (0-1)	79.43%	3.18%	0.63%
24mth:			
Uncertain (2)	10.24%	1.37%	0.37%
24mth:			
Not appropriate (≥3)	3.18%	1.10%	0.50%

Cross-sectionally, univariate relationships were observed between the proportion of children in GUiNZ with recommended TST and night wakings at 24 and 45 months of age and sociodemographic variables, as indicated by 95% confidence intervals not overlapping [23] (Supplementary Tables S1 and S2). At 24 months (Supplementary Table S1), a smaller proportion of Māori, Pacific, and Asian children had “appropriate” TST and night wakings compared to European/Other children. Increasing socioeconomic deprivation was associated with a decreasing percentage of children having “appropriate” TST and a larger proportion of children living in most deprived areas woke three or more times per night than children in least deprived areas. A greater proportion of children living in neighborhoods that were safe or did not have heavy traffic; in households with sufficient income to meet material needs, two parents or mothers in paid employment; and children who attended childcare, had very good or excellent health, or who usually spent two to less than five hours outside on weekdays had “appropriate” TST compared to their counterparts. A larger percentage of children living in rural areas, neighborhoods without heavy traffic, two-parent households or households with adequate income, and with excellent health usually woke an “appropriate” number of times at night.

At 45 months (Supplementary Table S2), a greater proportion of children in GUiNZ living in rural areas, households with two parents, or who had mothers in paid employment had “appropriate” TST. Less visual media use was associated with an increasing proportion of children having “appropriate” TST and night wakings.

In multivariable models, after controlling for all sociodemographic factors simultaneously, gender, ethnicity, NZDep, heavy traffic, and body size were significantly associated with sleep duration; and ethnicity, heavy traffic, rurality, material standard of living, household structure, and child health were significantly associated with night wakings, at 24 months (Supplementary Table S3). Prevalence estimates of children in NZ meeting or not meeting sleep duration and night waking guidelines at 24 months of age, after controlling for all sociodemographic variables (i.e. gender, ethnicity, NZDep, rurality, material standard of living, neighborhood safety, heavy traffic, household structure, maternal paid work, childcare, time outside week, general health, and body size) were calculated. Estimates are presented (Table 3) by gender, ethnicity, NZDep, and all other significant predictors from multivariable models i.e. heavy traffic and body size for sleep duration; and rurality, material standard of living, heavy traffic, and general health for night wakings.

**Table 3.** Adjusted^a^ prevalence estimates of children in NZ meeting or not meeting sleep duration and night waking guidelines at 24 months of age

Sociodemographic Variable	Sleep Variable		
	TST		
	Short % (95% HDI)^b^	Appropriate % (95% HDI)^b^	Long % (95% HDI)^b^
All predictors^a^	15.57 (14.90-16.27)	70.20 (69.29-71.09)	14.23 (13.58-14.91)
Gender:			
Boy	14.68 (14.02-15.35)	70.21 (69.31-71.11)	15.11 (14.44-15.80)
Girl*	16.54 (15.85-17.25)	70.17 (69.27-71.07)	13.29 (12.65-13.94)
Ethnicity:			
European/Other	10.22 (9.64-10.82)	70.95 (70.05-71.84)	18.83 (18.08-19.60)
Māori*	17.50 (16.77-18.25)	71.11 (70.21-72.00)	11.39 (10.79-12.02)
Pacific*	27.54 (26.68-28.41)	66.37 (65.44-67.29)	6.09 (5.64-6.57)
Asian*	22.91 (22.10-23.74)	69.14 (68.23-70.04)	7.95 (7.43-8.49)
NZDep quintile:			
1 (least deprived)	11.98 (11.36-12.61)	70.84 (69.94-71.73)	17.18 (16.46-17.92)
2	11.87 (11.27-12.50)	70.59 (69.69-71.48)	17.53 (16.81-18.28)
3	13.72 (13.07-14.39)	70.79 (69.89-71.68)	15.49 (14.81-16.20)
4	17.00 (16.29-17.73)	70.64 (69.74-71.53)	12.36 (11.74-13.00)
5* (most deprived)	23.38 (22.58-24.20)	68.13 (67.21-69.04)	8.49 (7.98-9.04)
Heavy traffic:			
Disagree	13.97 (13.32-14.64)	70.49 (69.59-71.38)	15.54 (14.86-16.24)
Agree (traffic)*	18.70 (17.97-19.45)	69.61 (68.70-70.51)	11.69 (11.09-12.31)
Body size:			
Healthy weight	14.34 (13.68-15.01)	70.50 (69.60-71.39)	15.16 (14.49-15.86)
Obese	16.26 (15.57-16.96)	69.71 (68.80-70.61)	14.03 (13.39-14.70)
Overweight*	17.80 (17.09-18.54)	69.86 (68.96-70.76)	12.33 (11.72-12.97)
Thin	21.93 (21.15-22.72)	68.07 (67.15-68.98)	10.00 (9.45-10.57)
	Night Wakings		
	Appropriate % (95% HDI)^b^	Uncertain % (95% HDI)^b^	Not appropriate % (95% HDI)^b^
All predictors^a^	84.58 (84.30-84.86)	11.61 (11.45-11.78)	3.81 (3.69-3.93)
Gender:			
Boy	84.17 (83.77-84.57)	11.86 (11.63-12.09)	3.97 (3.79-4.14)
Girl	85.01 (84.63-85.40)	11.35 (11.12-11.58)	3.64 (3.47-3.80)
Ethnicity:			
European/Other	87.52 (87.26-87.79)	9.83 (9.66-10.00)	2.65 (2.55-2.75)
Māori	82.78 (82.16-83.40)	12.74 (12.40-13.09)	4.48 (4.19-4.76)
Pacific	82.69 (82.01-83.38)	12.94 (12.53-13.36)	4.37 (4.09-4.65)
Asian*	76.94 (76.14-77.75)	16.05 (15.66-16.45)	7.00 (6.58-7.43)
NZDep quintile:			
1 (least deprived)	87.71 (87.23-88.19)	9.63 (9.33-9.93)	2.66 (2.47-2.84)
2	84.89 (84.32-85.47)	11.47 (11.13-11.82)	3.63 (3.39-3.88)
3	83.03 (82.32-83.73)	12.51 (12.12-12.90)	4.46 (4.13-4.79)
4	84.52 (83.91-85.12)	11.68 (11.32-12.04)	3.80 (3.54-4.06)
5 (most deprived)	82.21 (81.57-82.85)	13.11 (12.75-13.47)	4.68 (4.39-4.97)
Rurality:			
Rural	89.86 (89.31-90.42)	8.20 (7.82-8.58)	1.94 (1.75-2.12)
Urban*	84.02 (83.72-84.31)	11.98 (11.81-12.15)	4.01 (3.88-4.13)
Sociodemographic Variable	Sleep Variable		
	Night Wakings		
	Appropriate % (95% HDI)^b^	Uncertain % (95% HDI)^b^	Not appropriate % (95% HDI)^b^
Material standard of living:			
Not enough	79.84 (78.87-80.80)	14.47 (13.97-14.96)	5.70 (5.21-6.18)
Just enough	81.04 (80.53-81.55)	13.79 (13.52-14.07)	5.17 (4.92-5.41)
Enough*	86.11 (85.74-86.48)	10.71 (10.48-10.94)	3.18 (3.03-3.33)
More than enough*	89.16 (88.82-89.50)	8.75 (8.52-8.98)	2.09 (1.97-2.20)
Heavy traffic:			
Disagree	85.96 (85.65-86.26)	10.78 (10.60-10.97)	3.26 (3.14-3.39)
Agree (traffic)*	81.81 (81.29-82.33)	13.29 (13.00-13.58)	4.90 (4.66-5.15)
Household structure:			
Parent alone	87.08 (86.11-88.06)	10.11 (9.47-10.75)	2.81 (2.45-3.16)
Two parents	86.12 (85.84-86.40)	10.70 (10.52-10.87)	3.18 (3.07-3.30)
Parent/s with others*	79.57 (78.95-80.19)	14.60 (14.28-14.93)	5.83 (5.52-6.13)
General health:			
Poor	70.90 (66.94-74.87)	18.59 (16.94-20.24)	10.50 (8.12-12.89)
Fair	71.16 (69.16-73.16)	18.59 (17.73-19.45)	10.25 (9.06-11.44)
Good	80.48 (79.62-81.34)	14.14 (13.66-14.61)	5.38 (4.99-5.77)
Very good	83.54 (83.12-83.97)	12.37 (12.11-12.62)	4.09 (3.91-4.27)
Excellent*	87.24 (86.96-87.52)	10.02 (9.84-10.20)	2.74 (2.64-2.84)

*Note:* HDI=highest density interval; *Significant independent predictor in the Bayesian multivariate linear regression model (TST) or Bayesian negative binomial multiple regression model (night waking) (Supplementary Table C) ^a^Simultaneously controlling for gender, ethnicity, NZDep, rurality, material standard of living, neighborhood safety, heavy traffic, household structure, maternal paid work, childcare, time outside week, general health, and body size; ^b^95% HDI (unobserved parameters lie within the HDI limits with a certain probability, given the observed data) were computed based on 10,000 iterations and in some of the estimates the resulting intervals are narrow and must be interpreted with caution

As outlined in Table 3, at 24 months of age approximately 30% of children in NZ have a high probability of not meeting sleep duration guidelines and 15% have a high probability of falling outside of recommended night waking guidelines. An estimated 18% of Māori, 28% of Pacific and 23% of Asian children have a high probability of short sleep versus 10% of European/Other children, and 23% of Asian children have a high probability of “uncertain” or “not appropriate” night wakings versus 12% of European/Other children. Around 23% of children living in most deprived areas have a high probability of short sleep versus 12% of children in least deprived areas, 19% of children living near heavy traffic, and 18% of children who are overweight have a high probability of short sleep versus 14% of children not living in areas with heavy traffic or with healthy weight. Approximately 16% of children living in urban areas, 18% of children living in close proximity to heavy traffic, and 29% of children with poor or fair health have a high probability of not meeting night waking guidelines versus those in rural areas (10%), areas without heavy traffic (14%) or with excellent health (13%).

After controlling for all sociodemographic factors simultaneously in multivariable models, gender was significantly associated with TST; and ethnicity and visual media use with night wakings, at 45 months of age (Supplementary Table S4). Prevalence estimates of 45-month old children in NZ meeting or not meeting sleep guidelines, after controlling for all sociodemographic variables (i.e. gender, ethnicity, NZDep, rurality, household structure, maternal paid work, and visual media use) are presented by gender, ethnicity, and NZDep, plus visual media use for night waking (Table 4). Results indicate that an estimated 20% of children in NZ have a high probability of not meeting sleep duration guidelines and approximately 8% have a high probability of not meeting night waking guidelines. Approximately 90% of Māori, Pacific, and Asian children have a high probability of “appropriate” night wakings versus 93% of European/Other children. Around 1.6 to 1.7% of children who use visual media more than 2 hours per day have a high probability of waking 3 or more times a night versus 1% of children who usually use visual media less than 1 hour per day.

**Table 4.** Adjusted^a^ prevalence estimates of children in NZ meeting or not meeting sleep duration and night waking guidelines at 45 months of age

Sociodemographic Variable	Sleep Variable		
	TST		
	Short % (95% HDI)^b^	Appropriate % (95% HDI)^b^	Long % (95% HDI)^b^
All predictors^a^	14.06 (13.39-14.76)	80.49 (79.70-81.26)	5.44 (5.01-5.90)
Gender:			
Boy	12.75 (12.10-13.41)	81.16 (80.38-81.92)	6.09 (5.63-6.58)
Girl*	15.47 (14.76-16.19)	79.78 (78.98-80.56)	4.75 (4.35-5.19)
Ethnicity:			
European/Other	12.66 (12.01-13.32)	81.26 (80.48-82.02)	6.09 (5.63-6.57)
Māori	14.37 (13.69-15.07)	80.41 (79.61-81.18)	5.23 (4.80-5.68)
Pacific	15.95 (15.24-16.68)	79.49 (78.69-80.28)	4.56 (4.16-4.99)
Asian	17.78 (17.03-18.54)	78.35 (77.53-79.15)	3.88 (3.51-4.27)
NZDep quintile:			
1 (least deprived)	13.58 (12.92-14.26)	80.77 (79.98-81.54)	5.65 (5.21-6.12)
2	12.87 (12.22-13.54)	81.07 (80.28-81.83)	6.06 (5.61-6.55)
3	14.41 (13.73-15.11)	80.34 (79.55-81.12)	5.25 (4.82-5.70)
4	14.22 (13.54-14.91)	80.41 (79.62-81.18)	5.37 (4.94-5.83)
5 (most deprived)	15.58 (14.87-16.30)	79.70 (78.90-80.48)	4.72 (4.32-5.16)
	Night Wakings		
	Appropriate % (95% HDI)^b^	Uncertain % (95% HDI)^b^	Not appropriate % (95% HDI)^b^
All predictors^a^	91.67 (91.62-91.72)	7.01 (6.96-7.05)	1.32 (1.31-1.33)
Gender:			
Boy	91.98 (91.91-92.06)	6.77 (6.72-6.83)	1.24 (1.23-1.26)
Girl	91.35 (91.27-91.43)	7.25 (7.19-7.31)	1.40 (1.38-1.42)
Ethnicity:			
European/Other	93.37 (93.33-93.40)	5.73 (5.70-5.75)	0.91 (0.90-0.92)
Māori*	89.88 (89.82-89.95)	8.36 (8.31-8.41)	1.75 (1.73-1.77)
Pacific*	90.36 (90.28-90.45)	8.00 (7.94-8.07)	1.63 (1.61-1.66)
Asian*	89.60 (89.51-89.69)	8.56 (8.49-8.63)	1.84 (1.81-1.86)
NZDep quintile:			
1 (least deprived)	92.21 (92.11-92.32)	6.60 (6.52-6.68)	1.19 (1.16-1.21)
2	92.08 (91.97-92.20)	6.69 (6.61-6.78)	1.22 (1.19-1.25)
3	92.28 (92.16-92.40)	6.55 (6.46-6.64)	1.17 (1.14-1.20)
4	91.73 (91.60-91.85)	6.97 (6.88-7.07)	1.30 (1.27-1.33)
5 (most deprived)	90.26 (90.17-90.36)	8.07 (7.99-8.14)	1.67 (1.64-1.69)
Visual media:			
<1hr	93.11 (93.02-93.20)	5.91 (5.85-5.98)	0.98 (0.96-1.00)
1-<2hr	92.66 (92.59-92.72)	6.27 (6.22-6.32)	1.07 (1.06-1.09)
2-<3hr*	90.52 (90.42-90.62)	7.88 (7.81-7.95)	1.60 (1.58-1.63)
≥3hr*	90.12 (90.02-90.21)	8.18 (8.11-8.25)	1.71 (1.68-1.73)

HDI, highest density interval; *Significant independent predictor in the Bayesian multivariate linear regression model (TST) or Bayesian negative binomial multiple regression model (night waking) (Supplementary Table D) ^a^Simultaneously controlling for gender, ethnicity, NZDep, rurality, household structure, maternal paid work, and visual media use; ^b^95% HDI (unobserved parameters lie within the HDI limits with a certain probability, given the observed data) were computed based on 10,000 iterations and in some of the estimates the resulting intervals are narrow and must be interpreted with caution

We modeled TST and night waking trajectories from 24 to 45 months of age using a Bayesian growth curve approach (Table 5). In this modeling framework all children have a common intercept (representing TST, or average number of night wakings, at 24 months) and a common slope (representing the rate of change from 24 to 45 months). In addition, each child has their own random effect, which is their individual-specific intercept and slope which depends on child gender, ethnicity, and NZDep quintile, and were included as predictors in models simultaneously. We found that European/Other boys living in least deprived areas had an average TST at 24 months of 12.9 hours (global intercept), which decreased across time by 1.5 hours (global slope) from 24 to 45 months of age. Average TST was shorter by 5 minutes for girls than boys at 24 months but there was no difference in the rate of change over time by gender. At 24 months, Māori, Pacific, and Asian children’s TST was significantly shorter (by 26, 46, and 37 minutes respectively) and TST changed at a significantly slower rate across the two timepoints, compared to European/Other children (Supplementary Figure S1). This meant that European/Other children tended to sleep more at 24 months but their TST declined more over time, resulting in TST at 45 months being more similar across ethnic groups.

**Table 5.** Estimated trajectories of sleep durations and number of night wakings from 24 to 45 months of age for children in NZ modeled by gender, ethnicity and socioeconomic deprivation, reported as adjusted^a^ mean, standard deviation and 95% highest density intervals

	Intercept (24 months)			Slope (change from 24 months to 45 months)		
	Total sleep time:	Total sleep time:	Total sleep time:	Total sleep time:	Total sleep time:	Total sleep time:
	Mean (SD)	95% HDI	Significance	Mean (SD)	95% HDI	Significance
Global intercept	12.86 (0.04)	12.78 to 12.95	Significant	–1.54 (0.05)	–1.65 to –1.45	Significant
Gender						
Girl	–0.08 (0.03)	–0.15 to –0.02	Significant	0.01 (0.04)	–0.06 to 0.08	Non-significant
Ethnicity						
Māori	–0.43 (0.05)	–0.52 to –0.34	Significant	0.29 (0.06)	0.16 to 0.42	Significant
Pacific	–0.77 (0.06)	–0.88 to –0.66	Significant	0.72 (0.07)	0.59 to 0.84	Significant
Asian	–0.62 (0.05)	–0.72 to –0.52	Significant	0.30 (0.06)	0.19 to 0.42	Significant
NZDep						
quintile	0.002 (0.06)	–0.11 to 0.11	Non-significant	0.02 (0.07)	–0.10 to 0.15	Non-significant
2	–0.10 (0.06)	–0.21 to 0.03	Non-significant	0.13 (0.08)	–0.06 to 0.26	Non-significant
3	–0.17 (0.06)	–0.29 to –0.07	Significant	0.16 (0.07)	0.07 to 0.32	Significant
4	–0.36 (0.06)	–0.49 to –0.25	Significant	0.34 (0.08)	0.22 to 0.55	Significant
5						
	Night wakings: Mean (SD)	Night wakings: 95% HDI	Night wakings: Significance	Night wakings: Mean (SD)	Night wakings: 95% HDI	Night wakings: Significance
Global intercept	0.54 (0.05)	–0.70 to –0.51	Significant	0.62 (0.06)	–0.61 to –0.38	Significant
Gender						
Girl	0.96 (0.03)	–0.11 to –0.02	Non-significant	1.09 (0.04)	0.03 to 0.16	Significant
Ethnicity						
Māori	1.20 (0.05)	0.07 to 0.28	Significant	1.08 (0.09)	–0.08 to 1.08	Non-significant
Pacific	1.08 (0.07)	–0.06 to 0.21	Non-significant	1.14 (0.13)	–0.12 to 0.39	Non-significant
Asian	1.46 (0.06)	0.26 to 0.49	Significant	0.90 (0.10)	–0.26 to 0.12	Non-significant
NZDep quintile						
2	1.01 (0.06)	–0.10 to 0.13	Non-significant	1.08 (0.08)	–0.08 to 0.21	Non-significant
3	1.09 (0.06)	–0.03 to 0.20	Non-significant	1.03 (0.08)	–0.11 to 0.17	Non-significant
4	1.02 (0.06)	–0.09 to 0.13	Non-significant	0.99 (0.08)	–0.16 to 0.15	Non-significant
5	1.11 (0.06)	–0.004 to 0.22	Non-significant	0.93 (0.07)	–0.22 to 0.08	Non-significant

SD, standard deviation; HDI, highest density interval

^a^Models were adjusted for child gender, ethnicity and NZDep quintile simultaneously

The average number of night wakings for European/Other boys living in the least socioeconomically deprived areas was 0.5 (global intercept), which changed at a rate of 0.6 (global slope) resulting in average night wakings at 45 months of 0.3 (Table 5). There was no difference in the average number of night wakings at 24 months by gender but there was less change across time for girls, resulting in girls having slightly more night wakings at 45 months (girls = 0.48; boys = 0.46). Māori and Asian children tended to wake more at 24 months compared to European/Other children, however, there was no difference in the rate of change by ethnicity resulting in a slightly higher average number of night wakings at 45 months for Māori and Asian children (0.54) compared to European/Other children (0.42). No significant differences were found in night waking trajectories by NZDep.

## Discussion

This research makes a significant contribution to the literature on sleep health trajectories in early childhood [24], social determinants of child sleep health [25, 26], and sleep health inequities [27] due to the large and diverse sample utilized and the analytical techniques that have been employed. Findings suggest that a considerable and concerning proportion of young children in NZ are not meeting sleep health guidelines. Inequitable patterns of sleep health were observed by ethnicity as early as 24 months of age and a range of sociodemographic factors including socioeconomic deprivation, material standard of living, rurality, and heavy traffic were identified as determinants of sleep health in early childhood, thus highlighting impacts of wider societal, economic and physical environments on sleep. The use of novel statistical modeling enabled potential variability in each child’s sleep to be included in analyses and the prevalence of children in NZ meeting, or not meeting, sleep guidelines at 24 and 45 months of age to be estimated cross-sectionally. Results indicate that approximately 30 percent of two-year-olds and 20 percent of four-year-olds in NZ have a high probability of not meeting sleep duration guidelines, and approximately 15 percent of two-year-olds and 8 percent of four-year-olds in NZ have a high probability of not meeting sleep quality guidelines, as indicated by usually waking twice or more at night.

Cross-sectional analyses indicated that at 24 months of age, gender, ethnicity, area-level socioeconomic deprivation, heavy neighborhood traffic, and body size were independent predictors of how long children slept and ethnicity, rurality, material standard of living, heavy neighborhood traffic and general health were significant independent predictors of how often children woke at night. Fewer sociodemographic variables were available to investigate cross-sectionally at 45 months but, of those that were, gender was a significant independent predictor of sleep duration, and ethnicity and visual media use were significant independent predictors of night wakings.

Further research is needed to investigate mechanisms involved in sleep health inequities at such an early age. Socioeconomic deprivation was a significant risk factor, which mirrors previous international research [26, 28], although some findings to date have been mixed [29]. The association between deprivation and sleep that we observed may, in part, have been due to less sleep-conducive environments such as household crowding or bedrooms that are too cold, noisy, or light [30] or increased stress on children due to material hardship [31]. Ethnicity remained a significant predictor of TST and night wakings at 24 months and night wakings at 45 months after controlling for NZDep, which suggests that the sleep of Māori, Pacific, and Asian children was influenced by something over and above the impact of deprivation. One potential explanation is that child or parental experiences of interpersonal racism [32] negatively influenced child sleep via increased stress and subsequent difficulties initiating or maintaining sleep [33]. Māori and Pacific peoples are over-represented in areas with greatest socioeconomic deprivation in NZ [34], which is indicative of structural racism [32]. Māori and Pacific children may therefore be doubly disadvantaged when it comes to sleep health, which supports the need for action at the political level to tackle structural racism.

We were unable to elucidate mechanisms involved in relationships we identified between neighborhood-, household-, and child-level factors and sleep. However, children living in urban environments may have experienced difficulties sleeping due to noise, light, close proximity of housing, or less physical activity due to environments being less conducive to active play than rural areas, and heavy neighborhood traffic may have impacted on children’s sleep via noise, vibration, or light. Greater duration of visual media use being associated with more night wakings may have been due, in part, to the content viewed [35]. Being overweight and having poorer health was associated with shorter and poorer quality sleep at 24 months of age however, as these relationships were cross-sectional, longitudinal tracking of sleep and health is needed to better understand the direction and causality.

Longitudinally, descriptive statistics indicated that around one quarter of children in the GUiNZ study did not meet sleep duration and night waking guidelines at both 24 and 45 months of age. Growth curve models provided insight into the social patterning of trajectories of sleep health in NZ from 24 to 45 months of age, with differences identified in some instances by gender, ethnicity, and area-level deprivation. Girls tended to have shorter sleep at 24 months and, as the rate of change in sleep duration from 24 to 45 months did not differ by gender, also at 45 months of age compared to boys. Girls and boys tended to wake a similar number of times at 24 months of age but night wakings decreased at a slower rate for girls over time, suggesting greater improvement in sleep quality across this time period for boys. Māori, Pacific, and Asian children tended to have shorter sleep durations than those of European/Other children at 24 months, however sleep durations declined over time at a greater rate for European/Other children resulting in more similar sleep durations between ethnic groups by 45 months of age. The rate of change of night wakings did not differ by ethnicity and Māori and Asian children tended to wake more at 24 and 45 months of age compared to European/Other children. Children living in more socioeconomically deprived areas tended to sleep less at 24 months but had a slower rate of decline in duration over time, resulting in more similar sleep durations across socioeconomic groups by the time children were 45 months old.

These findings suggest that sleep duration and continuity may improve to some extent as children get older but because data were only available at two timepoints it is not yet known how sleep health trajectories may differ by gender, ethnicity, or socioeconomic deprivation over longer periods of time. By providing evidence that the disproportionate risk for poor sleep health for Māori children exists as early as 24 months of age, these findings build on existing research that Māori 3─4 year-olds are at a greater risk of poor sleep health than non-Māori 3─4 year-olds and that socioeconomic deprivation is a significant independent risk factor for poor sleep health in early childhood [9, 10]. It also extends current knowledge of sleep health inequities in NZ by providing evidence of Pacific and Asian children being at an increased risk of poor sleep health, and on international evidence of racial/ethnic disparities in sleep whereby 2─5 year-olds from minoritized racial/ethnic groups in the US are disproportionately burdened by poor sleep health [27].

A number of limitations must be acknowledged. All measures were maternally reported and parents have been shown to overestimate child sleep duration compared to objectively measured sleep [36]. However, as current sleep duration guidelines are based largely on evidence using parent-report [37] the subjective sleep duration variables used in this study are considered appropriate. It is also important to note that NSF guidelines, on which NZ guidelines are based, categorize sleep duration in five levels. We collapsed “inappropriate” and “may be appropriate” short or long sleep into single categories of “short” and “long” sleep, therefore children who are naturally and appropriately short or long sleepers may have been categorized as having inappropriate sleep durations. It is also not known whether NSF sleep guidelines, which include wide ranges of recommended sleep durations, are directly transferable to the NZ population and how meeting or not meeting these guidelines in early childhood relates to health in NZ. Future research is therefore warranted to examine relationships between “not recommended”, “may be appropriate” and “recommended” sleep durations and night wakings and indicators of child waking functioning, health, and development cross-sectionally and longitudinally.

Nonetheless, a strength of this study was the utilization of a large, demographically diverse dataset which enabled a greater understanding of relationships between sociodemographic factors and sleep across early childhood. While sleep data were only available at 24 and 45 months of age when this study was conducted, the longitudinal study design did allow changes in two measures of sleep health over time to be investigated and better understood. Findings inform the need for future research including ongoing monitoring and analysis of sleep and sociodemographic data, and the investigation of relationships between sleep health trajectories and mental and physical health outcomes and inequities, over time. Results, including that ethnic inequities in sleep health exist early in the life-course and the link between visual media use and sleep, highlight the importance of developing a range of culturally responsive and informative sleep resources for children and families that do not take a one-size-fits-all approach to supporting good sleep health. However of particular importance, results highlight the urgent need to tackle socio-political drivers of sleep health inequities, including structural racism, and the importance of taking a multi-level approach to supporting child sleep health as opposed to sleep health interventions being solely focused on behavioral sleep advice at the individual child-level [38].

In summary, not all children in NZ are meeting sleep duration and sleep quality recommendations in early childhood and inequities in sleep health exist as early as 24 months of age. Therefore, comprehensive, equity-based, multi-level interventions are needed to support equitable sleep health across early childhood.

## Supplementary Material

zpac002_suppl_Supplementary_Material

## Data Availability

The data underlying this article were accessed in accordance with the GUiNZ Data Access Protocol and cannot be shared by the authors.
