# Correction to: Who meets national early childhood sleep guidelines in Aotearoa New Zealand? A cross-sectional and longitudinal analysis

**DOI:** 10.1093/sleepadvances/zpac008

**Published:** 2022-03-21

**Authors:** D Muller, E Santos-Fernandez, J McCarthy, H Carr, T L Signal

**Affiliations:** 1 Sleep/Wake Research Centre, School of Health Sciences, College of Health, Massey University, Wellington, New Zealand; 2 Faculty of Science, School of Mathematical Sciences, Queensland University of Technology, Brisbane, Australia; 3 Ministry of Health, New Zealand

In the originally published version of this manuscript, there was an error in the first column of Table 4 whereby asterisks were placed in the wrong position: i.e., they were erroneously added next to the ‘Māori’ ‘Pacific’ and ‘Asian’ ethnicity and NZDep quintile ‘5’ categories for TST and erroneously omitted from the ‘Māori’ and ‘Pacific’ ethnicity and ‘2 - <3hr’ visual media categories for Night Wakings. These errors have been corrected.

