# Supplementary material for: Who meets national early childhood sleep guidelines in Aotearoa New Zealand? A cross-sectional and longitudinal analysis
Source: Sleep Adv. 2022 Jan 16;3(1):zpac002. doi: 10.1093/sleepadvances/zpac002 (PMC10104380; doi:10.1093/sleepadvances/zpac002)
Supplement: zpac002_suppl_Supplementary_Material [file zpac002_suppl_supplementary_material.docx]

**Who meets national early childhood sleep guidelines in Aotearoa New Zealand? A cross-sectional and longitudinal analysis**

Authors: Dr D Muller (ORCID 0000-0003-2497-8946)^1^, Dr E Santos-Fernandez^2^ (ORCID 0000-0001-5962-5417), Dr J McCarthy^3^, Dr H Carr^3^, Prof T L Signal (ORCID 0000-0002-3414-1982)^1^

Author affiliations: ^1^Sleep/Wake Research Centre, School of Health Sciences, College of Health, Massey University, New Zealand; ^2^ Faculty of Science, School of Mathematical Sciences, Queensland University of Technology, Australia; ^3^Ministry of Health, New Zealand

Corresponding author:

Dr Diane (Dee) Muller

Sleep/Wake Research Centre

School of Health Sciences

College of Health

Massey University

PO Box 756

Wellington 6140

Ph: +64 4 9793261

Email: [d.p.muller@massey.ac.nz](mailto:d.p.muller@massey.ac.nz)

**Supplementary Table S1**. Cross-sectional proportions (and 95% confidence intervals)^a^ of children in the GUiNZ study meeting sleep duration and night waking guidelines at 24 months of age, stratified by sociodemographic variables (n=6,308)

|  | Total Sleep Time | | | Night Wakings | | |
| --- | --- | --- | --- | --- | --- | --- |
| Sociodemographic Variable | Short  (<11hr)  % (95% CI) | Appropriate  (11-14hr)  % (95% CI) | Long  (>14hr)  % (95% CI) | Appropriate  (0-1 waking)  % (95% CI) | Uncertain  (2 wakings)  % (95% CI) | Not appropriate  (≥3 wakings)  % (95% CI) |
| Gender:  Girl  Boy | 12.76 (11.57-13.94)  11.02 (9.94-12.09) | 82.42 (81.07-83.77)  83.15 (81.87-84.44) | 4.82 (4.06-5.58)  5.83 (5.03-6.63) | 83.73 (82.42-85.04)  82.54 (81.24-83.84) | 11.97 (10.82-13.12)  12.30 (11.18-13.43) | 4.30 (3.58-5.02)  5.15 (4.40-5.91) |
| Ethnicity:  Māori  Pacific  Asian  European/Other | 16.26 (14.37-18.16)  25.39 (22.44-28.33)  12.33 (10.14-14.51)  5.49 (4.66-6.31) | 78.57 (76.46-80.68)  69.61 (66.49-72.72)  83.99 (81.55-86.43)  88.48 (87.32-89.64) | 5.17 (4.03-6.31)  5.01 (3.53-6.48)  3.69 (2.43-4.94)  6.03 (5.17-6.89) | 82.15 (80.18-84.12)  81.29 (78.65-83.93)  76.15 (73.32-78.99)  86.33 (85.09-87.58) | 12.06 (10.38-13.74)  13.59 (11.27-15.91)  17.40 (14.87-19.92)  9.92 (8.84-11.00) | 5.79 (4.59-6.99)  5.13 (3.63-6.62)  6.45 (4.82-8.09)  3.75 (3.06-4.44) |
| NZDep quintile:  1 (least deprived)  2  3  4  5 (most deprived) | 5.06 (3.78-6.34)  7.45 (5.93-8.97)  9.23 (7.52-10.95)  13.99 (12.05-15.92)  19.57 (17.60-21.55) | 89.43 (87.64-91.23)  86.59 (84.61-88.57)  85.10 (82.99-87.21)  82.05 (71.91-84.19)  74.74 (72.58-76.91) | 5.51 (4.17-6.84)  5.96 (4.59-7.33)  5.67 (4.30-7.04)  3.96 (2.87-5.05)  5.68 (4.53-6.84) | 85.35 (83.28-87.41)  84.66 (82.57-86.75)  83.09 (80.87-85.31)  82.30 (80.17-84.42)  80.75 (78.79-82.71) | 11.55 (9.68-13.41)  10.34 (8.57-12.11)  11.88 (9.97-13.80)  12.69 (10.84-14.55)  13.63 (11.92-15.34) | 3.11 (2.09-4.12)  5.00 (3.73-6.26)  5.03 (3.73-6.32)  5.01 (3.80-6.23)  5.62 (4.47-6.77) |
| Rurality:  Rural  Urban | 8.68 (6.23-11.13)  11.97 (11.12-12.81) | 84.22 (81.05-87.39)  82.84 (81.86-83.82) | 7.10 (4.86-9.34)  5.19 (4.62-5.77) | 87.38 (84.49-90.27)  82.66 (81.67-83.65) | 9.66 (7.09-12.24)  12.36 (11.50-13.21) | 2.96 (1.48-4.43)  4.98 (4.41-5.55) |
| Material standard of living:  Not enough  Just enough  Enough  More than enough | 15.72 (12.92-18.52)  14.54 (13.02-16.06)  11.31 (10.01-12.60)  6.39 (5.05-7.74) | 78.43 (75.26-81.59)  80.72 (79.03-82.42)  83.24 (81.71-84.77)  87.77 (85.96-89.57) | 5.86 (4.05-7.66)  4.73 (3.82-5.65)  5.46 (4.53-6.39)  5.84 (4.55-7.13) | 77.97 (74.78-81.15)  80.63 (78.93-82.33)  84.68 (83.20-86.15)  87.13 (85.29-88.98) | 15.56 (12.77-18.35)  13.53 (12.05-15.00)  11.35 (10.05-12.65)  9.47 (7.86-11.08) | 6.47 (4.58-8.36)  5.85 (4.83-6.86)  3.97 (3.17-4.77)  3.39 (2.40-4.39) |
| Neighborhood safety:  Disagree  Agree (safe) | 16.33 (13.25-19.42)  11.39 (10.57-12.21) | 78.04 (74.58-81.50)  83.33 (82.36-84.29) | 5.63 (3.70-7.55)  5.28 (4.70-5.86) | 81.85 (78.63-85.07)  83.22 (82.25-84.19) | 11.25 (8.61-13.89)  12.25 (11.40-13.10) | 6.90 (4.78-9.01)  4.53 (3.99-5.07) |

^a^Based on observational data and not adjusted for any other sociodemographic variables i.e. univariate relationships between each sociodemographic and sleep variable

Supplementary Table S1 continued:

|  | Total Sleep Time | | | Night Wakings | | |
| --- | --- | --- | --- | --- | --- | --- |
| Sociodemographic Variable | Short  (<11hr)  % (95% CI) | Appropriate  (11-14hr)  % (95% CI) | Long  (>14hr)  % (95% CI) | Appropriate  (0-1 waking)  % (95% CI) | Uncertain  (2 wakings)  % (95% CI) | Not appropriate  (≥3 wakings)  % (95% CI) |
| Neighborhood traffic:  Disagree  Agree (traffic) | 10.78 (9.84-11.73)  13.83 (12.36-15.29) | 84.00 (82.88-85.12)  80.57 (78.89-82.25) | 5.22 (4.54-5.90)  5.60 (4.63-6.58) | 84.17 (83.05-85.28)  81.18 (79.52-82.83) | 11.65 (10.67-12.63)  13.08 (11.65-14.51) | 4.18 (3.57-4.79)  5.74 (4.76-6.73) |
| Household structure:  Parent alone  Two parents  Parent/s + others | 16.09 (12.04-20.13)  9.70 (8.82-10.59)  16.08 (14.32-17.84) | 78.86 (74.37-83.36)  85.14 (84.07-86.21)  78.14 (76.16-80.12) | 5.05 (2.64-7.46)  5.16 (4.49-5.82)  5.78 (4.66-6.89) | 82.02 (77.79-86.25)  84.60 (83.52-85.68)  79.69 (77.77-81.61) | 11.04 (7.59-14.49)  11.46 (10.51-12.42)  14.00 (12.34-15.66) | 6.94 (4.14-9.74)  3.94 (3.35-4.52)  6.31 (5.15-7.48) |
| Maternal paid work:  Yes  No | 10.19 (9.16-11.22)  13.70 (12.46-14.94) | 85.38 (84.18-86.58)  79.93 (78.48-81.37) | 4.43 (3.73-5.13)  6.38 (5.50-7.26) | 84.09 (82.84-85.33)  82.05 (80.67-83.43) | 11.60 (10.51-12.69)  12.75 (11.55-13.95) | 4.31 (3.62-5.00)  5.20 (4.40-5.99) |
| Childcare:  Yes  No | 9.91 (8.92-10.90)  14.30 (13.00-15.60) | 85.64 (84.48-86.80)  79.25 (77.74-80.76) | 4.45 (3.77-5.14)  6.45 (5.53-7.36) | 84.29 (83.09-85.50)  81.66 (80.22-83.10) | 11.56 (10.51-12.62)  12.86 (11.61-14.11) | 4.14 (3.48-4.80)  5.48 (4.63-6.32) |
| Time outside week:  0-<1hr  1-<2hr  2-<3hr  3-<4hr  4-<5hr  ≥5hr | 18.02 (14.17-21.86)  12.92 (10.75-15.10)  9.98 (8.64-11.32)  10.73 (9.05-12.41)  11.23 (9.07-13.40)  13.98 (11.69-16.27) | 74.93 (70.59-79.28)  81.49 (78.97-84.01)  85.21 (83.62-86.80)  84.52 (82.56-86.48)  82.42 (79.81-85.02)  80.57 (77.95-83.18) | 7.05 (4.49-9.61)  5.59 (4.10-7.08)  4.81 (3.85-5.76)  4.75 (3.60-5.91)  6.35 (4.68-8.02)  5.45 (3.95-6.95) | 79.90 (75.88-83.91)  82.69 (80.24-85.15)  83.80 (82.15-85.45)  84.29 (82.32-86.27)  85.47 (83.06-87.88)  80.00 (77.36-82.64) | 12.79 (9.45-16.14)  12.05 (9.94-14.16)  11.60 (10.16-13.03)  11.42 (9.69-13.14)  9.89 (7.85-11.93)  15.80 (13.39-18.21) | 7.31 (4.70-9.92)  5.26 (3.81-6.71)  4.60 (3.66-5.54)  4.29 (3.19-5.39)  4.64 (3.20-6.08)  4.20 (2.88-5.53) |
| General health:  Poor  Fair  Good  Very good  Excellent | 28.12 (12.55-43.70)  16.91 (11.80-22.01)  15.51 (12.76-18.27)  11.87 (10.49-13.25)  10.64 (9.59-11.70) | 62.50 (45.73-79.27)  79.71 (74.23-85.19)  80.72 (77.72-83.72)  82.91 (81.30-84.51)  83.54 (82.27-84.80) | 9.38 (0.00-19.47)  3.38 (0.92-5.84)  3.77 (2.32-5.21)  5.22 (4.27-6.17)  5.82 (5.02-6.62) | 65.62 (49.17-82.08)  68.60 (62.28-74.92)  79.52 (76.45-82.59)  82.29 (80.66-83.92)  85.48 (84.27-86.68) | 6.25 (0.00-14.64)  18.84 (13.51-24.17)  13.86 (11.23-16.48)  12.58 (11.17-14.00)  11.13 (10.05-12.20) | 28.12 (12.55-43.70)  12.56 (8.05-17.07)  6.63 (4.73-8.52)  5.13 (4.19-6.07)  3.40 (2.78-4.01) |

^a^Based on observational data and not adjusted for any other sociodemographic variables i.e. univariate relationships between each sociodemographic and sleep variable

Supplementary Table S1 continued.

|  | Total Sleep Time | | | Night Wakings | | |
| --- | --- | --- | --- | --- | --- | --- |
| Sociodemographic Variable | Short  (<11hr)  % (95% CI) | Appropriate  (11-14hr)  % (95% CI) | Long  (>14hr)  % (95% CI) | Appropriate  (0-1 waking)  % (95% CI) | Uncertain  (2 wakings)  % (95% CI) | Not appropriate  (≥3 wakings)  % (95% CI) |
| Body size:  Thin  Healthy weight  Overweight  Obese | 16.81 (9.92-23.71)  8.17 (6.92-9.43)  13.56 (10.85-16.27)  12.07 (7.88-16.26) | 78.76 (71.22-86.30)  86.01 (84.42-87.60)  82.68 (79.68-85.68)  82.76 (77.90-87.62) | 4.42 (0.63-8.22)  5.81 (4.74-6.89)  3.76 (2.25-5.26)  5.17 (2.32-8.02) | 79.65 (72.22-87.07)  84.64 (82.99-86.30)  83.50 (80.56-86.44)  83.19 (78.38-88.00) | 17.70 (10.66-24.74)  10.59 (9.17-12.00)  12.42 (9.81-15.03)  14.22 (9.73-18.72) | 2.65 (0.00-5.62)  4.77 (3.79-5.75)  4.08 (2.52-5.65)  2.59 (0.54-4.63) |

^a^Based on observational data and not adjusted for any other sociodemographic variables i.e. univariate relationships between each sociodemographic and sleep variable

**Supplementary Table S2**. Cross-sectional proportions (and 95% confidence intervals)^a^ of children in the GUiNZ study meeting sleep duration and night waking guidelines at 45 months of age, stratified by sociodemographic variables (n=6,186)

|  | Total Sleep Time | | | Night Wakings | | |
| --- | --- | --- | --- | --- | --- | --- |
| Sociodemographic variable | Short  (<10hr)  % (95% CI) | Appropriate  (10-13hr)  % (95% CI) | Long  (>13hr)  % (95% CI) | Appropriate  (0-1 waking)  % (95% CI) | Uncertain  (2 wakings)  % (95% CI) | Not appropriate  (≥3 wakings)  % (95% CI) |
| Gender:  Girl  Boy | 6.57 (5.68-7.45)  5.87 (5.06-6.69) | 90.55 (89.50-91.60)  90.66 (89.65-91.67) | 2.88 (2.28-3.48)  3.47 (2.83-4.10) | 92.33 (91.37-93.28)  93.38 (92.52-94.24) | 6.00 (5.14-6.85)  5.31 (4.53-6.09) | 1.68 (1.21-2.14)  1.31 (0.92-1.71) |
| Ethnicity:  Māori  Pacific  Asian  European/Other | 8.56 (7.07-10.05)  10.43 (8.24-12.62)  9.47 (7.47-11.46)  2.74 (2.14-3.33) | 87.90 (86.16-86.84)  84.22 (81.61-86.84)  88.23 (86.03-90.43)  95.01 (94.22-95.81) | 3.54 (2.56-4.53)  5.35 (3.74-6.96)  2.31 (1.28-3.33)  2.25 (1.71-2.79) | 90.48 (88.92-92.04)  91.71 (89.74-93.69)  90.66 (88.67-92.64)  94.81 (94.00-95.62) | 7.16 (5.79-8.53)  6.68 (4.89-8.47)  7.77 (5.94-9.59)  4.09 (3.36-4.81) | 2.36 (1.55-3.17)  1.60 (0.70-2.50)  1.58 (0.73-2.43)  1.11 (0.73-1.49) |
| NZDep quintile:  1 (least deprived)  2  3  4  5 (most deprived) | 3.37 (2.36-4.38)  4.64 (3.39-5.88)  4.83 (3.51-6.15)  6.71 (5.14-8.29)  10.33 (8.70-11.96) | 94.33 (93.03-95.63)  93.36 (91.89-94.83)  92.61 (91.00-94.22)  90.29 (88.42-92.15)  84.58 (82.64-86.52) | 2.30 (1.46-3.14)  2.00 (1.17-2.83)  2.56 (1.59-3.53)  3.00 (1.92-4.07)  5.09 (3.91-6.27) | 93.76 (92.40-95.11)  93.09 (91.59-94.59)  93.30 (91.76-94.84)  92.77 (91.14-94.40)  91.02 (89.48-92.55) | 4.60 (3.42-5.78)  5.09 (3.79-6.39)  5.62 (4.20-7.03)  5.48 (4.04-6.91)  7.41 (6.01-8.81) | 1.64 (0.93-2.36)  1.82 (1.03-2.61)  1.08 (0.45-1.72)  1.76 (0.93-2.58)  1.57 (0.90-2.24) |
| Rurality:  Rural  Urban | 4.26 (2.56-5.96)  6.30 (5.63-6.97) | 92.96 (90.81-95.12)  90.60 (89.80-91.40) | 2.78 (1.39-4.16)  3.10 (2.62-3.58) | 93.89 (91.87-95.91)  92.60 (91.88-93.32) | 4.44 (2.71-6.18)  5.83 (5.18-6.47) | 1.67 (0.59-2.75)  1.57 (1.23-1.91) |
| Household structure:  Parent alone  Two parents  Parent/s + others | 8.25 (5.83-10.67)  4.89 (4.26-5.53)  9.72 (8.11-11.32) | 87.12 (84.18-90.07)  92.41 (91.62-93.19)  86.07 (84.20-87.95) | 4.63 (2.78-6.47)  2.70 (2.22-3.18)  4.21 (3.12-5.30) | 92.76 (90.48-95.04)  93.18 (92.44-93.93)  91.81 (90.33-93.30) | 5.63 (3.61-7.66)  5.35 (4.69-6.02)  6.66 (5.31-8.01) | 1.61 (0.50-2.72)  1.46 (1.11-1.82)  1.53 (0.86-2.20) |
| Maternal paid work:  Yes  No | 5.83 (5.06-6.59)  6.71 (5.75-7.68) | 91.69 (90.79-92.60)  89.12 (87.92-90.32) | 2.48 (1.97-2.99)  4.17 (3.40-4.94) | 93.17 (92.34-94.00)  92.48 (91.46-93.49) | 5.55 (4.80-6.30)  5.79 (4.89-6.69) | 1.28 (0.91-1.65)  1.74 (1.23-2.24) |

^a^Based on observational data and not adjusted for any other sociodemographic variables i.e. univariate relationships between each sociodemographic and sleep variable

Supplementary Table S2 continued.

|  | Total Sleep Time | | | Night Wakings | | |
| --- | --- | --- | --- | --- | --- | --- |
| Sociodemographic variable | Short  (<10hr)  % (95% CI) | Appropriate  (10-13hr)  % (95% CI) | Long  (>13hr)  % (95% CI) | Appropriate  (0-1 waking)  % (95% CI) | Uncertain  (2 wakings)  % (95% CI) | Not appropriate  (≥3 wakings)  % (95% CI) |
| Visual media use:  <1hr  1-<2hr  2-<3hr  ≥3hr | 3.81 (2.68-4.94)  4.43 (3.56-5.30)  6.05 (4.85-7.26)  10.88 (9.27-12.49) | 93.56 (92.11-95.01)  92.68 (91.58-93.78)  91.29 (89.86-92.71)  84.52 (82.65-86.39) | 2.63 (1.68-3.57)  2.89 (2.18-3.60)  2.66 (1.85-3.47)  4.60 (3.52-5.69) | 94.11 (92.72-95.50)  93.89 (92.88-94.91)  92.69 (91.37-94.00)  90.59 (89.07-92.10) | 4.53 (3.31-5.76)  4.85 (3.94-5.76)  5.65 (4.48-6.82)  7.67 (6.29-9.05) | 1.36 (0.68-2.04)  1.26 (0.79-1.73)  1.66 (1.02-2.31)  1.74 (1.07-2.42) |

^a^Based on observational data and not adjusted for any other sociodemographic variables i.e. univariate relationships between each sociodemographic and sleep variable

**Supplementary Table S3**. Associations between sociodemographic factors and sleep duration and night wakings at 24 months of age, reported as adjusted^a^ mean, standard deviation and 95% highest density intervals (n=6,308)

|  | Total Sleep Time | | | Night Wakings | | |
| --- | --- | --- | --- | --- | --- | --- |
|  | Mean (SD) | 95% HDI | Significance | Mean (SD) | 95% HDI | Significance |
| Intercept | 12.93 (0.42) | 12.15–13.77 | Significant | 0.65 (0.40) | -1.03–0.09 | NS |
| Gender:  Boy  Girl | Baseline  -0.14 (0.05) | -0.25– -0.04 | Significant | Baseline  0.97 (0.05) | -0.12–0.97 | NS |
| Ethnicity:  European/other  Māori  Pacific  Asian | Baseline  -0.33 (0.07)  -0.71 (0.10)  -0.63 (0.08) | -0.48– -0.19  -0.90– -0.51  -0.80– -0.47 | Significant  Significant  Significant | Baseline  1.10 (0.07)  1.07 (0.09)  1.33 (0.07) | -0.04–0.23  -0.11–0.24  0.14–0.43 | NS  NS  Significant |
| NZDep quintile:  1  2  3  4  5 | Baseline  0.07 (0.08)  0.01 (0.09)  -0.10 (0.09)  -0.27 (0.09) | -0.09–0.23  -0.15–0.18  -0.27–0.06  -0.45– -0.08 | NS  NS  NS  Significant | Baseline  1.11 (0.08)  1.12 (0.08)  1.01 (0.08)  1.06 (0.09) | -0.04–0.26  -0.04–0.26  -0.14–0.17  -0.11–0.23 | NS  NS  NS  NS |
| Rurality:  Rural  Urban | Baseline  -0.13 (0.10) | -0.31–0.06 | NS | Baseline  1.27 (0.10) | 0.04–0.43 | Significant |
| Material standard of living:  Not enough  Just enough  Enough  More than enough | Baseline  -0.08 (0.10)  0.04 (0.10)  0.11 (0.11) | -0.28–0.12  -0.16–0.24  -0.11–0.32 | NS  NS  NS | Baseline  0.96 (0.09)  0.81 (0.09)  0.74 (0.10) | -0.21–0.12  -0.37– -0.03  -0.50– -0.11 | NS  Significant  Significant |
| Neighborhood safety:  Disagree  Agree (safe) | Baseline  -0.16 (0.11) | -0.37–0.05 | NS | Baseline  0.96 (0.09) | -0.22–0.15 | NS |
| Neighborhood traffic:  Disagree  Agree (traffic) | Baseline  -0.18 (0.06) | -0.29– -0.06 | Significant | Baseline  1.15 (0.05) | 0.04–0.24 | Significant |
| Household structure:  Parent alone  Two parents  Parent/s with others | Baseline  0.14 (0.14)  0.05 (0.14) | -0.13–0.40  -0.22–0.32 | NS  NS | Baseline  1.19 (0.14)  1.38 (0.14) | -0.09–0.47  0.06–0.63 | NS  Significant |
| Maternal paid work:  Yes  No | Baseline  0.01 (0.06) | -0.11–0.13 | NS | Baseline  1.00 (0.06) | -0.12–0.12 | NS |

*Note:* SD=standard deviation; HDI=highest density interval; NS=non-significant
^a^Adjusted for all sociodemographic variables listed in the table simultaneously

Supplementary Table S3 continued.

|  | Total Sleep Time | | | Night Wakings | | |
| --- | --- | --- | --- | --- | --- | --- |
|  | Mean (SD) | 95% HDI | Significance | Mean (SD) | 95% HDI | Significance |
| Intercept | 12.93 (0.42) | 12.15–13.77 | Significant | 0.65 (0.40) | -1.03–0.09 | NS |
| Childcare:  No  Yes | Baseline  0.05 (0.06) | -0.07–0.17 | NS | Baseline  1.07 (0.06) | -0.05–0.18 | NS |
| Time outside week:  0–<1hr  1–<2hr  2–<3hr  3–<4hr  4–<5hr  ≥5hr | Baseline   - 1. (0.13)   0.19 (0.12)  -0.13 (0.12)  0.11 (0.13)  -0.08 (0.13) | -0.24–0.25  -0.04–0.42  -0.37– -0.10  -0.15–0.36  -0.34–0.01 | NS  NS  NS  NS  NS | Baseline  1.08 (0.11)  1.02 (0.10)  1.04 (0.11)  0.96 (0.12)  1.16 (0.12) | -0.12–0.29  -0.18–0.22  -0.17–0.25  -0.28–0.18  -0.09–0.37 | NS  NS  NS  NS  NS |
| General health:  Poor  Fair  Good  Very good  Excellent | Baseline  -0.24 (0.37)  -0.12 (0.35)  0.08 (0.34)  0.14 (0.34) | -0.97–0.44  -0.83–0.52  -0.61–0.72  -0.55–0.77 | NS  NS  NS  NS | Baseline  1.04 (0.23)  0.75 (0.22)  0.69 (0.21)  0.59 (0.21) | -0.40–0.51  -0.70–0.16  -0.77–0.07  -0.92– -0.09 | NS  NS  NS  Significant |
| Body size:  Healthy weight  Obese  Overweight  Thin | Baseline  0.04 (0.10)  -0.17 (0.07)  -0.21 (0.13) | -0.15–0.23  -0.30– -0.04  -0.48–0.06 | NS  Significant  NS | Baseline  0.84 (0.10)  0.96 (0.06)  1.04 (0.12) | -0.37–0.02  -0.16–0.08  -0.20–0.26 | NS  NS  NS |

*Note:* SD=standard deviation; HDI=highest density interval; NS=non-significant
^a^Adjusted for all sociodemographic variables listed in the table simultaneously

**Supplementary Table S4**. Associations between sociodemographic factors and sleep duration and night wakings at 45 months of age, reported as adjusted^a^ mean, standard deviation and 95% highest density intervals (n=6,186)

|  | Total Sleep Time | | | Night Wakings | | |
| --- | --- | --- | --- | --- | --- | --- |
|  | Mean (SD) | 95% HDI | Significance | Mean (SD) | 95% HDI | Significance |
| Intercept | 11.35 (0.13) | 11.09–11.62 | Significant | 0.37 (0.12) | -1.23– -0.76 | Significant |
| Gender:  Boy  Girl | Baseline  -0.14 (0.04) | -0.23– -0.05 | Significant | Baseline  1.05 (0.04) | -0.03– -0.13 | NS |
| Ethnicity:  European/other  Māori  Pacific  Asian | Baseline  -0.06 (0.06)  -0.12 (0.08)  -0.21 (0.07) | -0.18–0.07  -0.29–0.04  -0.35– -0.07 | NS  NS  NS | Baseline  1.25 (0.05)  1.21 (0.07)  1.28 (0.06) | 0.12–0.33  0.05–0.32  0.13–0.36 | Significant  Significant  Significant |
| NZDep quintile:  1  2  3  4  5 | Baseline  0.05 (0.07)  -0.02 (0.07)  0.02 (0.07)  -0.01 (0.08) | -0.08–0.18  -0.16–0.11  -0.13–0.17  -0.17–0.14 | NS  NS  NS  NS | Baseline  0.98 (0.06)  0.95 (0.07)  0.95 (0.07)  0.99 (0.07) | -0.14–0.11  -0.18–0.08  -0.18–0.08  -0.14–0.12 | NS  NS  NS  NS |
| Rurality:  Rural  Urban | Baseline  -0.04 (0.07) | -0.19–0.11 | NS | Baseline  1.01 (0.07) | -0.13–0.14 | NS |
| Household structure:  Parent alone  Two parents  Parent/s with others | Baseline  0.07 (0.09)  0.01 (0.10) | -0.12–0.25  -0.19–0.21 | NS  NS | Baseline  1.07 (0.08)  1.03 (0.08) | -0.09–0.22  -0.14–0.20 | NS  NS |
| Maternal paid work:  Yes  No | Baseline  -0.03 (0.05) | -0.12–0.06 | NS | Baseline  0.97 (0.04) | -0.11–0.05 | NS |
| Visual media use:  <1hr  1–<2hr  2–<3hr  ≥3hr | Baseline  -0.01 (0.06)  -0.05 (0.07)  -0.08 (0.08) | -0.13–0.11  -0.19–0.08  -0.23–0.07 | NS  NS  NS | Baseline  1.03 (0.06)  1.16 (0.06)  1.15 (0.07) | -0.08–0.15  0.03–0.28  0.01–0.27 | NS  Significant  Significant |

*Note:* SD=standard deviation; HDI=highest density interval; NS=non-significant
^a^Adjusted for all sociodemographic variables listed in the table simultaneously

**Supplementary Figure S1**. Trajectory of average TST from 24 to 45 months of age, by child ethnicity


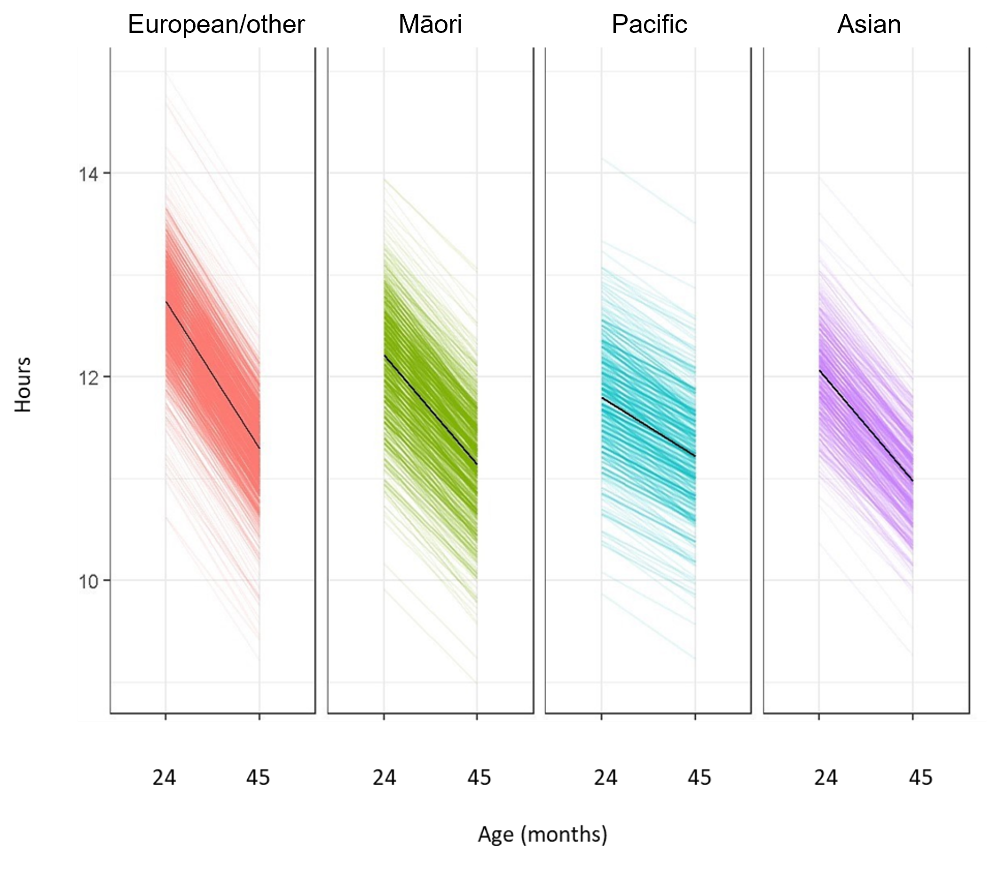


**Supplementary Material S1.**

This file contains some of the rjags models used for the Bayesian regression analyses of the total sleep time and the number of wakes.

**TST**

modelTSTall = '

model{

for(i in 1:N){

TST[i] ~ dnorm(mu_0[i], prec_b)

mu_0[i] = b0 + b_sex[sex[i]] +

b_eth[eth[i]] + b_NZDep2006[NZDep2006[i]] +

b_rurality[rurality_[i]] +

b_material[material_[i]] + b_safe[safe_[i]] +

b_noise[noise_[i]] + b_household[household_[i]] +

b_paidwork[paidwork_[i]] + b_childcare[childcare_[i]] +

b_outside_week[outside_week_[i]] + #b_outside_weekend[outside_weekend_[i]] +

b_health[health_[i]] + b_bmi_category[bmi_category_[i]]

TST_pred[i] ~ dnorm(mu_0[i], prec_b)

TST_not_app_low[i] <- ifelse(TST_pred[i] < 11, 1, 0)

TST_not_app_high[i] <- ifelse(TST_pred[i] > 14, 1, 0)

}

prec_b ~ dgamma(0.01,0.01)

sigma_b <- 1 / sqrt(prec_b)

#priors

b0 ~ dnorm(0, 1/10^3)

b_sex[1] <- 0 # baseline is boy. Set to 0

b_sex[2] ~ dnorm(0, 1/10^3)

b_eth[1] <- 0 # eth Other (baseline) to 0

for(j in 2:4){

b_eth[j] ~ dnorm(0,1/10^3)

}

b_NZDep2006[1] <- 0 # NZDep 1 (baseline) to 0

for(k in 2:5){

b_NZDep2006[k] ~ dnorm(0,1/10^3)

}

b_rurality[1] <- 0 # baseline is rural

b_rurality[2] ~ dnorm(0, 1/10^3)

b_material[1] <- 0 #

for(k in 2:4){

b_material[k] ~ dnorm(0,1/10^3)

}

b_safe[1] <- 0 # baseline is 1 Disagree . 2 Agree

b_safe[2] ~ dnorm(0, 1/10^3)

b_noise[1] <- 0 # baseline is 1 Disagree . 2 Agree

b_noise[2] ~ dnorm(0, 1/10^3)

b_household[1] <- 0 # 1 parent_alone

for(k in 2:3){

b_household[k] ~ dnorm(0,1/10^3)

}

b_paidwork[1] <- 0 # baseline is 1 = Yes ; 2 = No

b_paidwork[2] ~ dnorm(0, 1/10^3)

b_childcare[1] <- 0 # baseline is 1 = Yes ; 2 = No

b_childcare[2] ~ dnorm(0, 1/10^3)

b_outside_week[1] <- 0 #

for(k in 2:6){

b_outside_week[k] ~ dnorm(0,1/10^3)

}

b_health[1] <- 0

for(k in 2:5){

b_health[k] ~ dnorm(0,1/10^3)

}

b_bmi_category[1] <- 0

for(k in 2:4){

b_bmi_category[k] ~ dnorm(0,1/10^3)

}

}

'

**Wake at night**

# #"app" = '0',"app" = '1', "unc" = '2',"not_app"= '>=3'

modelWAKEall = '

model{

for(i in 1:N){

wake[i] ~ dnegbin(p[i], size)

p[i] <- size / (size + mu_0[i])

log(mu_0[i]) <- b0 + b_sex[sex[i]] +

b_eth[eth[i]] + b_NZDep2006[NZDep2006[i]] +

b_rurality[rurality_[i]] +

b_material[material_[i]] + b_safe[safe_[i]] +

b_noise[noise_[i]] + b_household[household_[i]] +

b_paidwork[paidwork_[i]] + b_childcare[childcare_[i]] +

b_outside_week[outside_week_[i]] + #b_outside_weekend[outside_weekend_[i]] +

b_health[health_[i]] + b_bmi_category[bmi_category_[i]]

wake_pred[i] ~ dnegbin(p[i], size)

wake_unc[i] <- ifelse(wake_pred[i] == 2, 1, 0)

wake_not_app[i] <- ifelse(wake_pred[i] >= 3, 1, 0)

}

#priors

size ~ dunif(0.001, 1000)

b0 ~ dnorm(0, 1/10^3)

b_sex[1] <- 0 # baseline is boy. Set to 0

b_sex[2] ~ dnorm(0, 1/10^3)

b_eth[1] <- 0 # eth Other (baseline) to 0

for(j in 2:4){

b_eth[j] ~ dnorm(0,1/10^3)

}

b_NZDep2006[1] <- 0 # NZDep 1 (baseline) to 0

for(k in 2:5){

b_NZDep2006[k] ~ dnorm(0,1/10^3)

}

b_rurality[1] <- 0 # baseline is rural

b_rurality[2] ~ dnorm(0, 1/10^3)

b_material[1] <- 0 #

for(k in 2:4){

b_material[k] ~ dnorm(0,1/10^3)

}

b_safe[1] <- 0 # baseline is 1 Disagree . 2 Agree

b_safe[2] ~ dnorm(0, 1/10^3)

b_noise[1] <- 0 # baseline is 1 Disagree . 2 Agree

b_noise[2] ~ dnorm(0, 1/10^3)

b_household[1] <- 0 # 1 parent_alone

for(k in 2:3){

b_household[k] ~ dnorm(0,1/10^3)

}

b_paidwork[1] <- 0 # baseline is 1 = Yes ; 2 = No

b_paidwork[2] ~ dnorm(0, 1/10^3)

b_childcare[1] <- 0 # baseline is 1 = Yes ; 2 = No

b_childcare[2] ~ dnorm(0, 1/10^3)

b_outside_week[1] <- 0 #

for(k in 2:6){

b_outside_week[k] ~ dnorm(0,1/10^3)

}

b_health[1] <- 0

for(k in 2:5){

b_health[k] ~ dnorm(0,1/10^3)

}

b_bmi_category[1] <- 0

for(k in 2:4){

b_bmi_category[k] ~ dnorm(0,1/10^3)

}

}

'
